# Extensive myocardial calcifications: a systematic literature review of a rare pathological phenomenon

**DOI:** 10.3389/fcvm.2024.1367467

**Published:** 2024-07-29

**Authors:** Fabiola B. Sozzi, Eleonora Gnan, Andrea Faggiano, Francesco Giangiacomi, Laura Iacuzio, Ciro Canetta, Gloria Santangelo, Marco Pisaniello, Armand Eker, Stefano Carugo

**Affiliations:** ^1^ Department of Cardio-Thoracic-Vascular Diseases, Foundation IRCCS Ca’ Granda Ospedale Maggiore Policlinico, Milan, Italy; ^2^ Department of Clinical Sciences and Community Health, University of Milan, Milan, Italy; ^3^ Centre Cardiothoracique, CCM, Monaco City, Monaco; ^4^ High Care Internal Medicine Unit, Foundation IRCCS Ca’ Granda Ospedale Maggiore Policlinico, Milan, Italy

**Keywords:** myocardial calcification, intra-myocardial calcifications, dystrophic calcification, metastatic calcification, myocardial infarction, endomyocardial fibrosis, computed tomography, cardiac magnetic resonance

## Abstract

**Introduction:**

Myocardial calcifications (MC) represent a relatively rare pathological process, which may accompany different cardiovascular conditions and can be broadly categorized as dystrophic or metastatic. Myocardial infarction (MI) has been traditionally regarded as the main cause of MC overall; however, no updated comprehensive data on the relative incidence of different forms of MC is available. The purpose of this systematic review of the literature is to analyze the currently available evidence on MC in terms of pathophysiology, diagnosis, and clinical presentation.

**Methods and results:**

A total of 241 studies including a total of 368 patients affected by extensive MC were included in the final review. The majority of patients (69.8%) presented with dystrophic MC. Endomyocardial fibrosis (EMF) represents the single most common etiology of MC (24.2%), while sepsis/acute systemic inflammatory syndrome (SIRS) and chronic kidney disease were identified as the second and third most common causes respectively. The relative incidence of etiologies also varies across the years, with MI being more represented before 1990, and sepsis/SIRS becoming the single most common cause of MC after 1990. Multimodality imaging was used in the work-up of MC in 42.7% of cases. The most commonly employed imaging modality overall was echocardiography (51.9%), while after 1990 computed tomography scan became the most widely used tool (70.1%).

**Conclusion:**

The present systematic review provides new insights into the pathophysiology of MC. Previously thought to be mainly a consequence of ischemic heart disease, our data indicate that other diseases, namely EMF and sepsis/SIRS, are indeed the main conditions associated with MC. The importance of multimodality imaging in the work-up of MC is also highlighted.

## 1 Introduction

Myocardial calcifications (MC) represent a relatively rare and often underrecognized pathological process, which may accompany different cardiovascular conditions.

MC can be broadly subdivided into two main groups according to their etiology: metastatic and dystrophic MC (1). The latter constitute the sequelae of local cardiac cell injury and necrosis, while metastatic MC can arise in both normal or diseased myocardium and are associated with abnormal calcium homeostasis and systemic conditions. Moreover, significant myocardial extension of valvular or pericardial calcifications can occur.

Dystrophic calcifications resulting from extensive myocardial infarction (MI) have been traditionally considered the most common type of MC. In recent years, however, advancements in the timing and quality of interventions for ischemic heart disease have led to a reduction in the incidence of extensive transmural myocardial necrosis. This, in turn, may imply a decline in the occurrence of dystrophic, post-infarction MC. At present, however, no systematic review of the literature providing updated insights into the relative incidence of different forms of MC is available.

The diagnosis of MC is challenging, and a thorough evaluation with the aid of multimodality imaging is often necessary to ascertain their etiology and clinical impact. Furthermore, current management of MC mostly relies on anecdotal experience stemming from single case reports or small case series, and no standardized, evidence-based approach exist.

The purpose of this systematic review is to analyze the currently available evidence on MC in terms of pathophysiology, diagnosis, and clinical presentation.

## 2 Materials and methods

The present systematic review was carried out according to the Preferred Reporting Items for Systematic Review and Meta-Analyses (PRISMA) standards. Pertinent literature was systematically scrutinized to identify all studies examining extensive MC. Isolated calcifications of the valvular apparatus (e.g., mitral annular calcifications or aortic valve calcifications), not significantly extending into the myocardial tissue, are not within the scope of this systematic literature review. Study designs comprised case reports, case series, short communications, retrospective and prospective studies and letters to the editors.

The PubMed, OVID-MEDLINE, and Cochrane library databases were systematically analysed to search articles published from inception to 30 June 2023. Studies were identified by crossing the following terms in title, abstract, and keywords: “myocardial calcification”, “intra-myocardial calcification”, “extensive calcification” “dystrophic calcification” and “metastatic calcification”. Two authors (AF and FG) assessed all titles and abstracts retrieved with the search. When there was an agreement on a specific record, the full text of the study was analysed by both reviewers to establish eligibility according to the inclusion criteria mentioned below. A third reviewer (FS) resolved disagreements on study judgments. Data extraction was performed by two reviewers (AF and FG) and independently checked by another reviewer (FS).

The main inclusion criteria were: (I) articles published in peer-reviewed journals; no unpublished or grey literature was searched; (II) studies providing data on living or dead human patients affected by extensive intra-myocardial calcifications. Specific exclusion criteria were: (I) studies involving foetal and neonatal calcifications; (II) studies involving patients affected by congenital heart disease, (III) reviews and editorials were excluded, but examined for potential additional references.

## 3 Results

**Figure 1:**
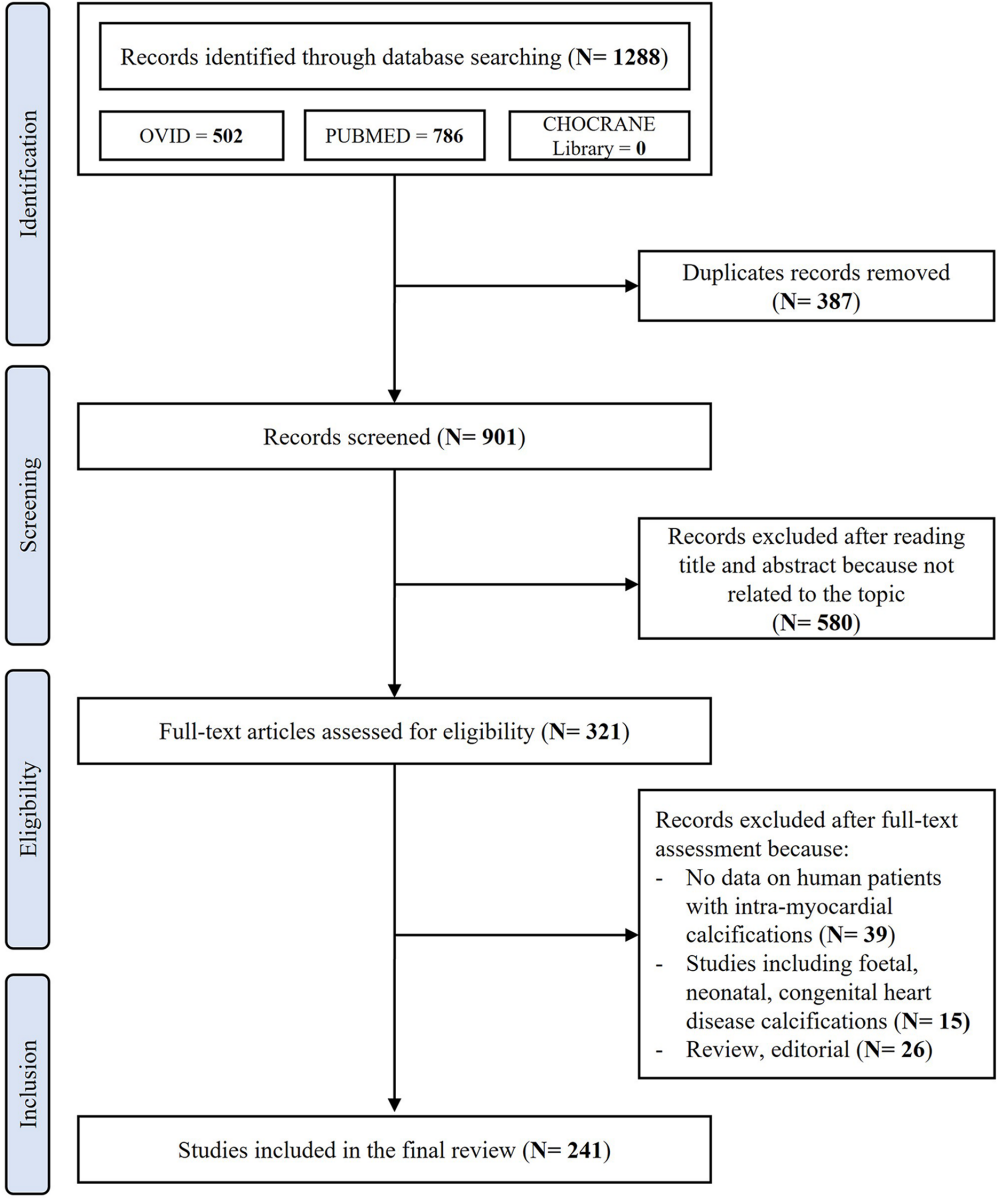
PRISMA flowchart showing the search strategy and manuscripts selection process.

After removing duplicates, the initial literature search identified 901 papers. After the initial screening of titles and abstracts, 580 studies were excluded as they were not related to the topic. Therefore, 321 studies were reviewed; of these, 39 did not report data on living or dead human patients affected by extensive MC, 26 were reviews or editorial articles and 15 were articles including foetal, neonatal calcifications or patients affected by congenital heart disease. Thus, a total of 241 studies including a total of 368 human patients affected by extensive MC were included in the final review (Figure 1). A complete list of the included studies is available as Supplementary Material.

**Figure 2:**
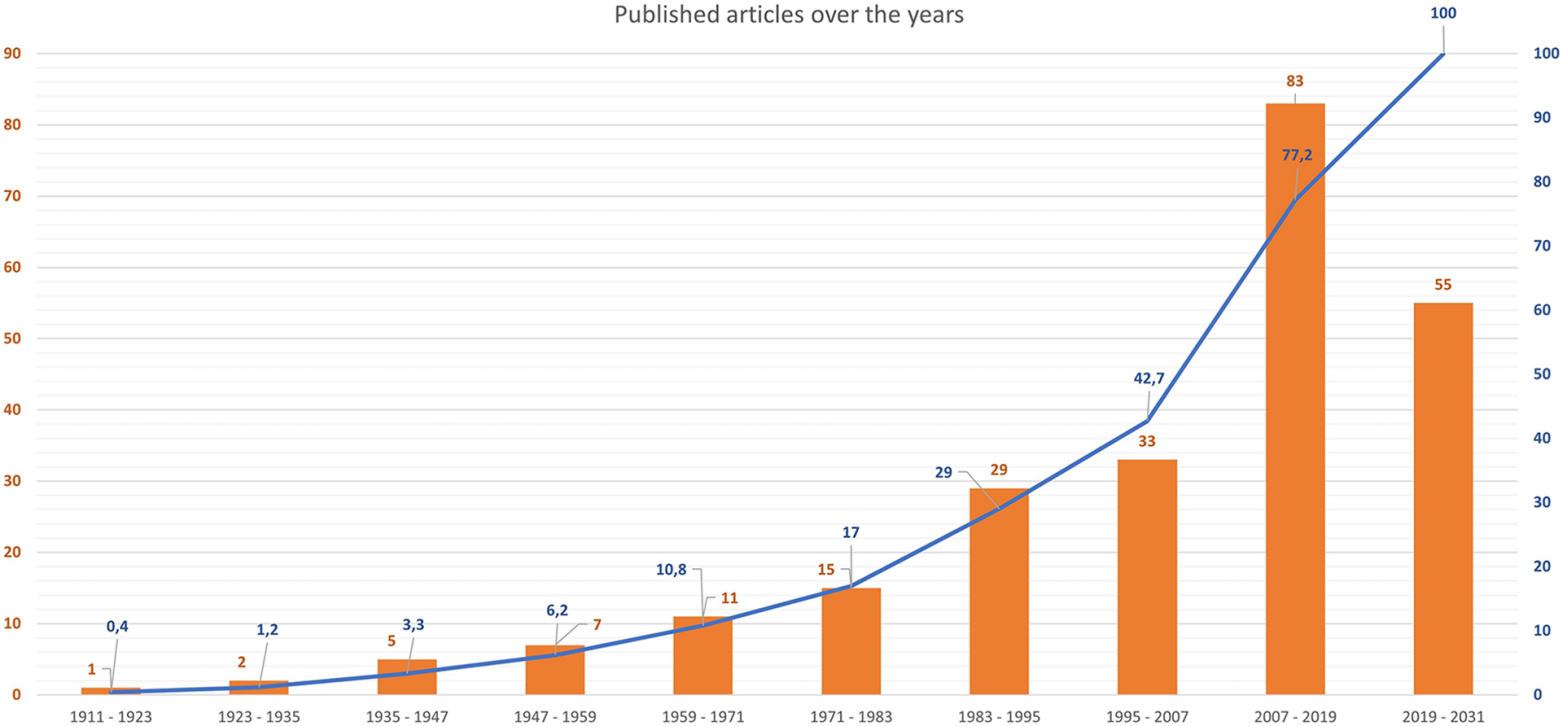
Timeline of published articles on MC. Bars show absolute numbers of published articles, while the overlay line in blue shows cumulative percentages (%).

Figure 2 shows the timeline of published articles on MC.

### 3.1 Clinical presentation

Age at presentation was reported in 279 cases (75.8%), and mean age was 45.9 ± 20.7 years. Overall, 167 patients (45.4%) were males, 114 females (31%), while no information regarding sex was available for 87 patients (23.6%).

MC affected the left ventricle only in most patients (*n* = 229, 62.2%), were confined to the right ventricle in 53 patients (14.4%) and showed biventricular distribution in 26 (7.1%).

A total of 37 patients (19.1%) with MC experienced sudden cardiac death (SCD), while shock or requirement of catecholamine support at presentation were reported in 62 patients (16.8%), irrespective of the cause of MC. *Ex*tracorporeal membrane oxygenation (ECMO) was used in 10 patients (2.7%), while 18 (4.9%) had a history of cardiac surgery.

### 3.2 Etiology

The majority of patients (*n* = 257, 69.8%) presented with dystrophic MC, while 73 subjects (19.8%) were diagnosed with metastatic MC. In a minority of patients (*n* = 6, 1.6%), both metastatic and dystrophic potential components coexisted. Finally, no clear etiology was identified or reported in the remaining 32 patients (8.7%).

Endomyocardial fibrosis (EMF) represents the single most common etiology of dystrophic MC and of the whole population (*n* = 89, 24.2%). EMF was associated with schistosomiasis in 7 cases (7.8%).

Information about age and sex was available for 42 out of 89 patients (47.2%) with EMF; mean age was 44 years and 25 (59.5%) were males. In these patients, MC involved predominantly the right ventricle in 40 patients (44.9%), the left ventricle in 36 (40.4%), and had biventricular distribution in 6 patients (6.7%).

The second and third most commonly reported causes of MC were sepsis/systemic inflammatory response syndrome (SIRS) and chronic kidney disease (CKD), affecting 50 (13.6%) and 44 (12%) patients respectively.

MI was recognized as the main cause of dystrophic MC in 34 patients (9.2%), but it is described as being at least a contributing factor in 43 cases (11.7%).

Other relatively common etiologies of dystrophic MC include myocarditis (*n* = 16, 4.3%), post-cardiac transplant MC (*n* = 11, 3%), hypertrophic cardiomyopathy (*n* = 9, 2.4%) and left ventricular non-compaction/dilated cardiomyopathy (LVNC/DCM, *n* = 3, 0.81%).

Metastatic MC not associated with CKD and resulting from disorders such as hyperparathyroidism, rickets or oxalosis were reported in 21 patients (5.7%).

A total of 19 patients (5.2%) were considered to have MC of multifactorial etiology. Finally, etiology of MC was either not recognized or not reported in 50 cases (13.6%). The complete list of etiologies with their relative frequency is presented in Table 1.

**Table 1:** Etiologies of myocardial calcifications.

Etiologies of myocardial calcifications (MC)	Total: 368
Male (%)	167 (45.4%)
Endomyocardial fibrosis (EMF)	89 (24.2%)
Sepsis/systemic inflammatory response syndrome (SIRS)	50 (13.6%)
Chronic kidney disease (CKD)	44 (12.0%)
Myocardial infarction (MI)	34 (9.2%)
Other disorders of calcium homeostasis	21 (5.7%)
Multifactorial	19 (5.2%)
Myocarditis	16 (4.3%)
Miscellaneous	12 (3.3%)
Cardiac transplant	11 (3%)
Myocardial extension of mitral annular calcification (MAC)	10 (2.7%)
Hypertrophic cardiomyopathy (CM)	9 (2.4%)
Left ventricular non compaction/dilated cardiomyopathy (LVNC/DCM)	3 (0.8%)
Unknown	50 (13.6%)

### 3.3 Distribution of etiologies according to year of publication

**Figure 3:**
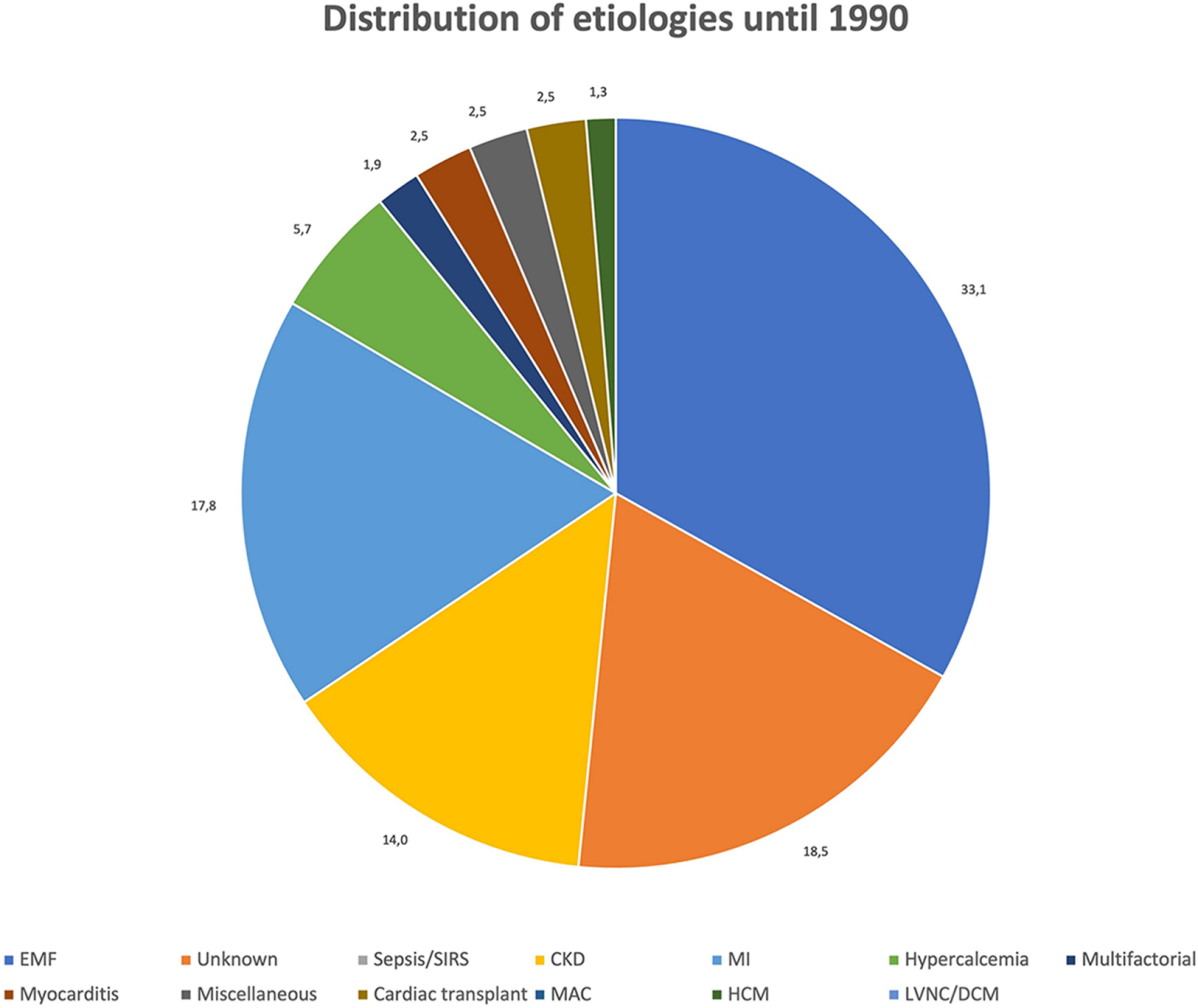
Distribution of etiologies until 1990. Values expressed as percentages (%).

**Figure 4:**
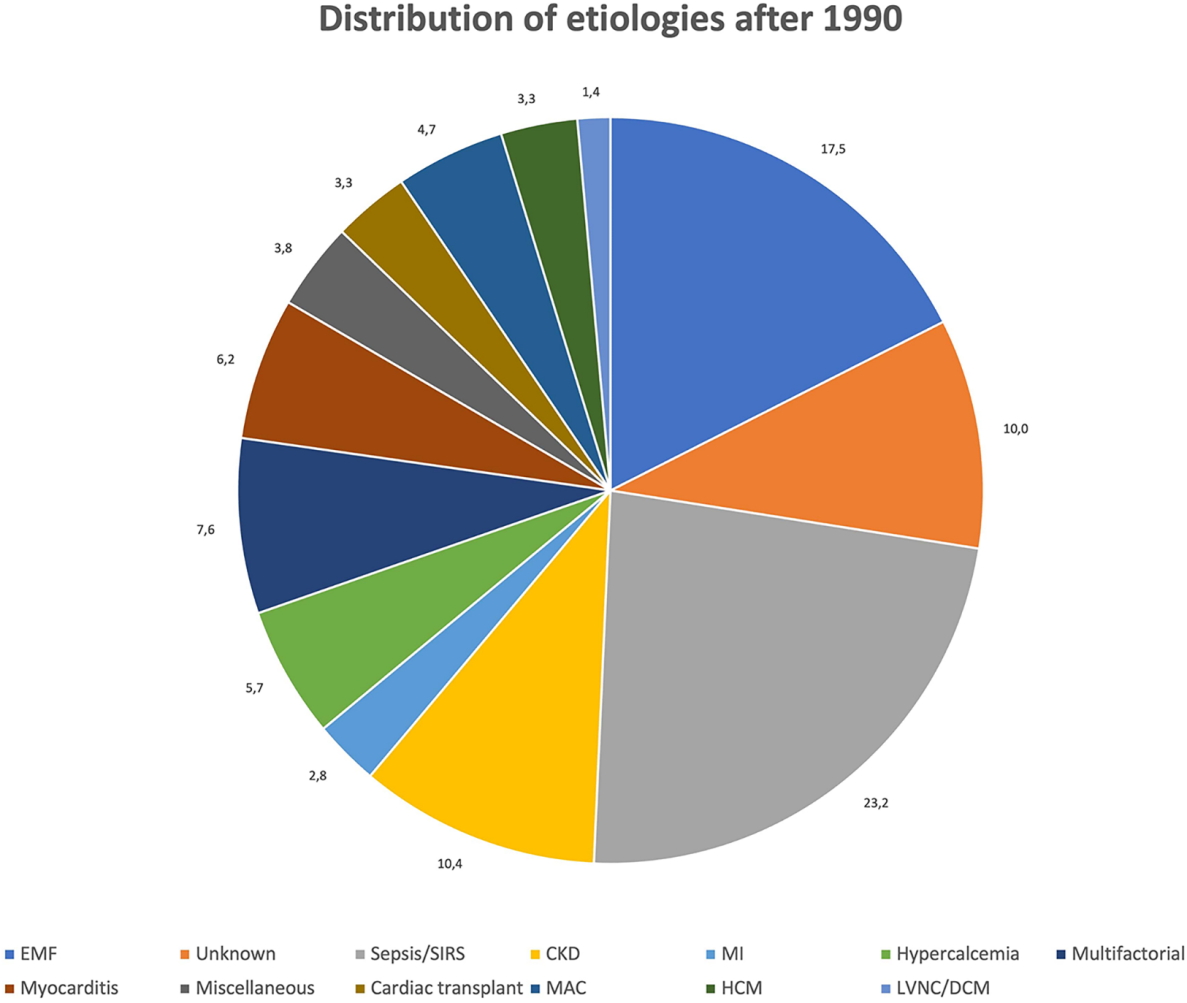
Distribution of etiologies after 1990. Values expressed as percentages (%).

Figures 3, 4 show the relative distribution of the various etiologies underlying MC according to whether the year of publication was before or after 1990.

Until 1990: A total of 169 cases of MC (45.9%) were published between 1911 and 1990, most of which (*n* = 109, 64.5%) were dystrophic MC, 2 (1.2%) resulted from both dystrophic and metastatic mechanisms, and 32 (18.9%) were classified as purely metastatic MC. As in the whole population, EMF appears to be the main cause of MC also in the period 1911–1990 (*n* = 64, 37.9%). Differently from what observed in the general population, the second most frequent cause of MC reported in the past was MI (*n* = 28, 16.6%), while there are no reports on MC secondary sepsis/SIRS, with the exception of a single case of MC secondary to a localized myocardial abscess (0.6%). CKD remains the third most commonly reported etiology of MC (*n* = 22, 13%).

After 1990: Data about 197 patients (53.5%) were published between 1991 and 2023. Dystrophic MC remain the most common form (148, 75.1%), while 4 patients (2%) had mixed dystrophic-metastatic etiology and 39 (19.8%) had purely metastatic MC. Among publications spanning from 1991 onward, sepsis/SIRS is by far the most frequent cause of MC, with a total of 49 cases (24.9%) identified. EMF (*n* = 25, 12.7%) and CKD (*n* = 22, 11.2%) are the second and third most frequent cause of MC after 1990, while only 6 cases (3%) of MC resulting from MI have been identified. On the contrary, other causes of dystrophic MC are more represented between 1991 and 2023 than in previous years, such as myocarditis (*n* = 13, 6.6%), hypertrophic cardiomyopathy (HCM, *n* = 7, 3.6%), cardiac transplant (*n* = 7, 3.6%) and LVNC/DCM (*n* = 3, 1.5%).

### 3.4 Use of imaging modalities according to year of publication

Diagnostic work-up of MC included the use of imaging modalities in 246 cases (66.8%). The most employed methodology was echocardiography (*n* = 191, 51.9%), followed by computed tomography (CT) scan (*n* = 157, 42.7%) and chest radiography (*n* = 135, 36.7%). Finally, a minority of patients (*n* = 43, 11.7%) received a cardiac magnetic resonance examination (CMR). Use of at least two imaging modalities was reported for 158 patients (42.9%), the most common combinations being echocardiography + chest radiography (*n* = 58, 15.8%) and echocardiography plus CT scan (*n* = 46, 12.5%).

Chest radiography, when performed, had a false negative rate of 13/135 (9.6%), while echocardiography showed a false negative rate of 10/191 (5.2%).

Autoptic examination was carried out in 156 patients (42.4%), and MC were diagnosed solely on the basis of autopsy, without the use of imaging, in 73 patients (19.8%).

Before 1991, the most commonly adopted imaging methods were chest radiography (76/169 patients, 45%) and echocardiography (64/169 patients, 37.9%). Only 17 patients (10.1%) received a CT scan and no patient received a CMR before 1991, while autopsy was performed in 76 cases (45%).

From 1991 to 2023, instead, CT scan had become the most frequently used method (138/197 patients, 70.1%), followed by echocardiography (127/197 patients, 64.5%). On the contrary, only a minority of patients received a chest radiography (76/197 patients, 38.5%) and 43 (21.8%) received a CMR. Finally, autopsy was performed in 80 patients (40.6%).

**Figure 5:**
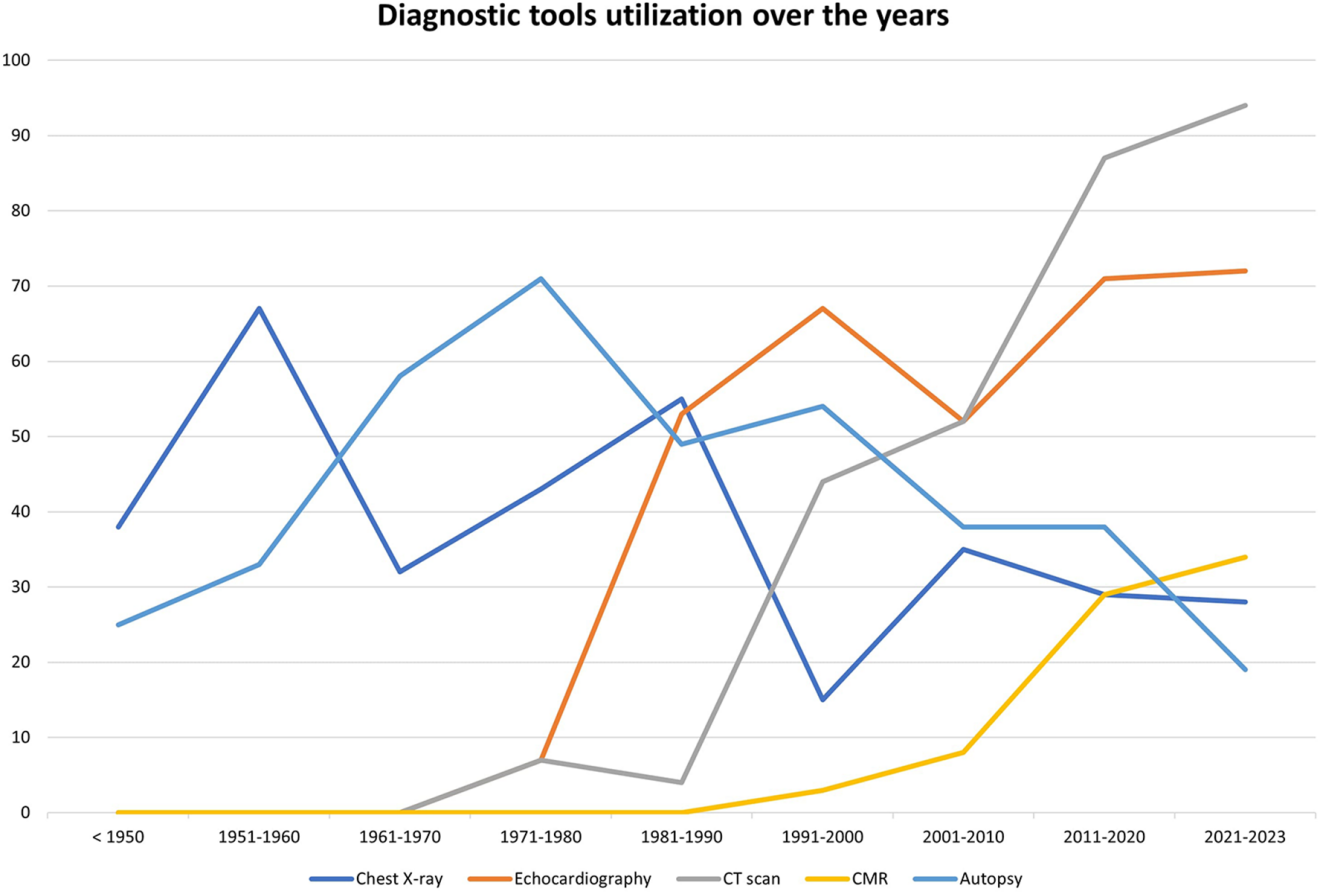
Trends of diagnostic tool utilization over the years.

Figure 5 shows the trends of diagnostic tool utilization over the years.

## 4 Discussion

The purpose of this systematic review is to discuss the relevant features of MC and to provide an updated picture of their etiologies, clinical presentations, and imaging characteristics.

MC can occur in two main forms depending on the mechanism by which they arise and their underlying etiology. In accordance to what previously reported by other authors (1), dystrophic MC are by far the most commonly encountered type of MC (68.9%), while metastatic MC are found in 19.8% of patients.

This different prevalence remains more or less stable across decades, even if the relative distribution of specific etiologies varies throughout the years. In particular, while in the past EMF followed by MI and CKD appeared to be the most common causes of MC, after 1991 sepsis/SIRS becomes the most frequently reported etiology, followed by EMF and CKD.

In the following paragraphs we individually discuss the main causes of MC and their relevant features.

### 4.1 Endomyocardial fibrosis

According to our systematic review of the literature, EMF is the most frequently described condition associated with MC, reported in 24.2% of cases, of which 7.8% had concomitant schistosomiasis. EMF is an idiopathic form of restrictive cardiomyopathy, which primarily affects young individuals of low socio-economic status in tropical and subtropical regions of the globe. Its cause is unknown and probably multifactorial, with infection (mostly parasitic), autoimmunity, genetic predisposition, diet and environmental exposures all recognized as potential contributors (2). The hallmark of the disease is ventricular obliteration by means of progressive fibrosis of the endocardium and subendocardial myocardium in the apex and inflow tract of the right ventricle, the left ventricle, or both. Initial stages of the disease are usually characterized by eosinophilic inflammation, vasculitis and necrosis of the affected cardiac tissue. This is followed by mural thrombi formation and subsequent fibrotic replacement. Moreover, calcifications are common in advanced stages (3). Our analysis reveals that MC associated with EMF involved almost equally the RV or the LV, while in a minority of patients had biventricular distribution.

### 4.2 Sepsis and systemic inflammatory response syndrome

The second most represented group of MC includes critically ill patients (*n* = 50, 13.6%), most commonly presenting with sepsis (*n* = 38, 10.3%). Isolated cases of MC in intensive care unit patients that were treated with catecholamines or were affected by SIRS due to miscellaneous causes including tumor lysis syndrome (4), rhabdomyolysis (5), and TAFRO (thrombocytopenia, anasarca, fever, renal insufficiency or reticulin fibrosis, organomegaly) syndrome (6) have also been described. The vast majority of these patients clinically presented with shock, and some of them received ECMO support during hospital stay (7).

Notably, sepsis/SIRS appear to be the most commonly reported cause of MC after 1991 (24.9% of cases), while no report antecedent to 1990 of MC associated with systemic infection or SIRS exists. The only case of MC secondary to an infection published before that year, namely in 1975, involves a case of MC formed at the site of an old, localized myocardial abscess (8).

The pathogenesis of MC in the context of sepsis is poorly understood. According to Bower et al. (9), sepsis is associated with a generalized inflammatory response that also involves the myocardium and leads to myocyte necrosis through several mechanisms, including mitochondrial injury, cellular edema and microvascular dysfunction. Extensive cardiomyocyte necrosis, in turn, may facilitate the formation of dystrophic MC. Another possible contributing factor to the myocardial injury in septic patients is the prolonged administration of catecholamines (10).

### 4.3 Myocardial infarction

In the past, MI has been reported as the most frequent cause of MC (1) in general, and of dystrophic MC in particular. According to our systematic review of the literature, however, MC secondary to MI seem to be less common than previously thought, being reported in 11.7% of patients overall.

This may be the result, at least in part, of improved efficacy of reperfusion and of their prompter implementation, which have reduced the incidence of large, transmural infarcts and, possibly, of subsequent MC formation in necrotic areas. In support of this hypothesis is the fact that while between 1911 and 1990 MI is reported as being the cause of MC in 16.6% of patients, this percentage decreases to 3% from 1991 onwards.

The largest case series of MC associated with MI was published in 1987 by Roberts et al. (11). Of 21 patients, 19 (90%) were males and mean age was 63 years, while mean age at first clinically apparent MI was 51 years. At autoptic gross examination, MC were often part of a left ventricular aneurysm (57%), the LV was consistently eccentrically hypertrophied in all patients, and 86% had evidence of obstructive disease in more than one coronary arteries.

Finally, our systematic review shows that 9 out of 43 patients with MI also presented with other conditions that might have contributed to the formation of MC, such as CKD. This supports the hypothesis that, in patients with MI or other local processes of tissue injury, disorders of calcium homeostasis represent a predisposing and facilitating factor for MC formation.

Other conditions that have emerged, especially in recent years, as possible causes of dystrophic MC include cardiomyopathies (12, 13), myocarditis (14) and cardiac transplant (15).

### 4.4 Metastatic calcifications

Metastatic calcifications represent the consequence of a systemic process associated with hypercalcemia and/or abnormalities of calcium homeostasis. In this context, crystal deposition can occur both in healthy or diseased tissue. Metastatic MC are most commonly associated with CKD (16, 17), which results in phosphate retention and secondary hyperparathyroidism and, according to the present analysis, was the cause MC in 13.6% of all cases. Other less frequent causes of metastatic MC include hyperparathyroidism of any cause (18), bone destruction or increased bone turnover as seen in rickets (19), and oxalosis (20). These, taken together, are responsible for 5.7% of all MC cases present in the literature.

### 4.5 Imaging findings

There are no standardized imaging features available to classify specific subtypes of intra-myocardial calcifications. Its diagnosis, evaluation of their possible etiology, and the clinical implications often mandates the use of multiple imaging techniques (42.7% of the cases according to our analysis) (21), which include chest radiography, echocardiography, CT scan and CMR.

Chest x-ray and echocardiography provide limited assessment. Chest radiography is a rapid and inexpensive tool that might be of use in the detection of MC, which was used in 36.7% of all cases and 45% of cases occurring after 1991 according to the current literature review. MC will appear as areas of increased density that may be difficult to precisely localize, and according to our analysis it presents a false negative rate of 9.6%.

Echocardiography was the most commonly used method of detection of MC overall (51.9%). It might be a useful and cost-effective first-line technique, showing an increased echodensity with posterior acoustic shadowing (1). Extensive shadowing or poor echocardiographic windows due to patient's characteristics, however, may limit the diagnostic accuracy of the technique, which according to our analysis presented a false negative rate of 5.2%. In addition to identification of MC, echocardiography provides information about cardiac systolic and diastolic function, which are often compromised in the context of MC.

High-quality cardiac CT scan currently represents the gold-standard test for the non-invasive detection of MC. Given its high spatial resolution, CT scan is the optimal modality to correctly identify, localize and define the extension of MC, which appear as areas of increased density as measured in Hounsfield Units. Our analysis shows that CT scan was increasingly implemented in recent times, and in the years 1991–2023 it has become the most frequently used tool to detect MC (70.1% of the cases).

Finally, CMR might also be used to characterize MC, which will show low signal on both T1- and T2-weighted images, with no evidence of LGE within the calcified areas but with possible LGE in the surrounding, necrotic myocardium (1, 21, 22). CMR with LGE and native T1/T2 mapping has the unique ability to provide a differential diagnosis in the whole spectrum of myocardial diseases, allowing tissue characterization and hemodynamic assessment.

**Figure 6:**
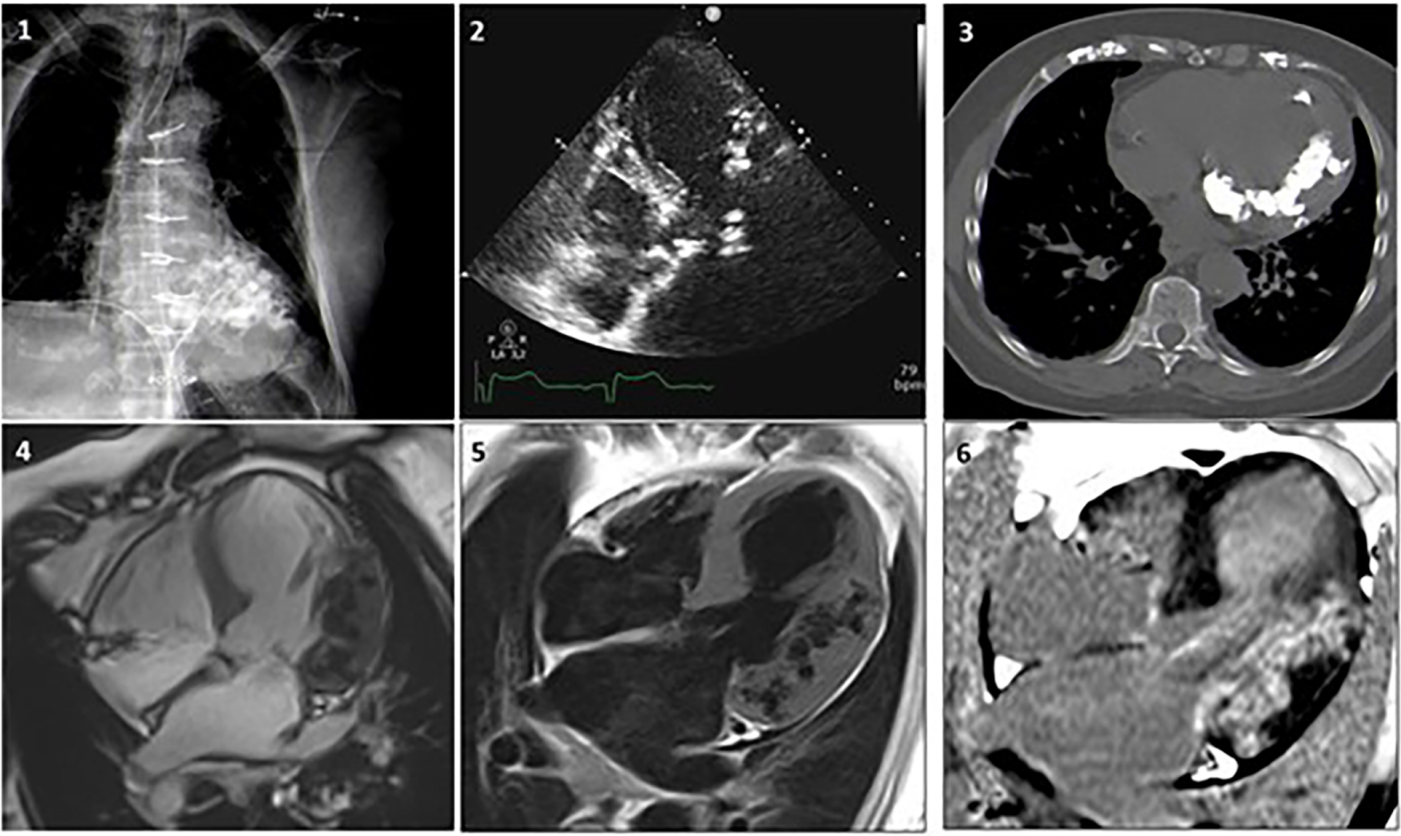
Case 1. A woman, 80 year-old, with CAD underwent revascularization through coronary artery bypass grafiting. (**1**) Chest x-ray showing a diffuse hypodense lobulated area in the left ventricle. (**2**) Echocardiogram revealing diffuse aortic and mitral calcifications with mild stenosis, marked septum and antero-lateral asymmetric hypertrophy, with extensive antero-lateral calcifications. (**3**) Non-contrast CT MPR axial-view presents widespread calcifications in the left ventricular wall extended to the mitral-aortic annulus and both coronary arteries. (**4**) CMR SSFP 4-chamber view showing lateral and antero-lateral wall thickening with areas of intra-myocardial low signal, surrounded by normal myocardium. Coronary angiography documented significant disease that required revascularization. (**5**) FSE T1 weighted-spin-echo4-chamber view is confirming the localization of lateral and anterolateral wall thickening with areas of low-signal. (**6**) After contrast gadolinium injection an extensive area of intra-myocardial hypersignal (hyperenhancement) is shown in the lateral wall. Adapted with permission from Sozzi et al. (21), licensed under CC BY 4.0.

In Figure 6, a clinical case from Sozzi et al (21), shows the additional diagnostic role of cardiac CT and CMR for evaluation of MC is shown. An 80-year-old woman with known CAD, with a history of coronary artery bypass surgery underwent chest x-ray, echocardiography, cardiac CT and CMR to ascertain the etiology of her MC. In this case the multimodality imaging analysis excluded previous MI, myocarditis and calcium-phosphate disorders. A dystrophic etiology was considered. The pathological mechanism for dystrophic calcifications is related to calcium deposition in any dead and dying myocardial tissues.

### 4.6 Clinical implications

The clinical implications of MC are often difficult to be ascertained. MC might be a direct contributor to the clinical presentation or merely a bystander and a marker of disease. Most likely, the truth lies somewhere in between. As described by some authors, it is plausible that the presence of MC may be at least partly responsible for systolic dysfunction and wall motion abnormalities (23, 24), increased myocardial stiffness and restrictive physiology (25, 26), and arrhythmias leading to SCD (27, 28).

In a study by Alyesh and colleagues (29), MC visualized by preprocedural CT scan were strongly associated with postinfarction ventricular tachycardia (VT) independent of other factors, and corresponded to nonexcitable tissue. Areas adjacent to MC represented the targets for mapping and ablation in >1/3 of patients with postinfarction VT.

Other authors, however, have questioned the hypothesis that calcifications are directly responsible for cardiac dysfunction and suggest a more passive, bystander role. In a case of MC associated with sepsis reported by Simonson and colleagues (30), serial CT examinations demonstrated rapid development of MC over a 2-week period. The patient's global systolic function worsened during the septic episode but improved to normal levels after resolution of the infection, although regional wall motion abnormalities persisted. In contrast, MC remained unchanged at a follow-up CT scan performed after 18 months. Several other cases of myocarditis or sepsis have been reported, in which the MC persisted after resolution of the acute episode and despite recovery of LVEF (14, 31, 32).

Regarding the behaviour of MC during follow-up, most reports unfortunately do not address the issue. While some describe medium- to long-term persistence of MC (14, 30), reports of at least partial resolution of MC over time also exist (33, 34). Zaidi and colleagues, for instance, described the case of a 6-year-old girl with MC due to secondary hyperparathyroidism, which significantly reduced in size after adequate vitamin D and calcium supplementation (35). Finally, Kimura and colleagues reported a case of fulminant myocarditis that required mechanical circulatory support and finally cardiac transplantation, in which the MC completely disappeared within 3 years after repeated CT examinations, despite the persistence of severe biventricular dysfunction (36).

### 4.7 Strengths and limitations

In the present work, we provide for the first time a systematic review of the available data on MC. Considering that most publications on the topic consist of case reports or small case series, it is of considerable value to finally have access to a uniform and comprehensive overview of this rare phenomenon. Among the strengths of our manuscript is certainly the description of the different causes of MC in relation to the year of publication, and the in-depth analysis of multimodal imaging used to diagnose them.

However, we also recognize some limitations. First, the nonrandomized nature and the limited number of patients included in the selected articles do not allow us to draw any firm conclusion regarding the frequency of MC in the general population and in the various disorders associated with them. Second, the analysis of the frequencies of the different etiologies of MC should also be looked at with a critical eye. For instance, the notion that EMF and sepsis/SIRS represent the leading causes of MC, and that conversely MI, historically considered the most common etiology, is less prevalent than expected, might be at least partly due to a publication bias. Third, many articles had missing data that precluded a more robust analysis. For instance, no information regarding sex was available for 23.6% of patients, and etiology of MC was either not recognized or not reported in 13.6%. Finally, many case reports are based on autopsy findings of MC alone, or otherwise do not provide patient follow-up. This makes it difficult to draw conclusions regarding the clinical implications of MC.

## 5 Conclusions

This systematic literature review provides an updated picture of etiologies, clinical presentations, and imaging characteristics of MC. In particular, it shows how the relative distribution of etiologies changes according to year of publication. There are no standardized imaging features available to classify specific subtypes of MC. Knowledge of the potential etiologies associated with MC and their imaging patterns are important to provide a concise and accurate differential diagnosis and to establish their clinical impact.

## Data Availability

The original contributions presented in the study are included in the article/**Supplementary Material**, further inquiries can be directed to the corresponding author.
