# Supplementary material for: Extensive myocardial calcifications: a systematic literature review of a rare pathological phenomenon
Source: Front Cardiovasc Med. 2024 Jul 29;11:1367467. doi: 10.3389/fcvm.2024.1367467 (PMC11317411; doi:10.3389/fcvm.2024.1367467)
Supplement: Supplementary file 1 [file Datasheet1.docx]

Supplementary Material

# List of all included articles

| **Author** | **Year** | **DOI** |
| --- | --- | --- |
| Sozzi | 2022 | 10.1111/echo.15357 |
| Segura | 2011 | 10.1016/j.carpath.2010.08.003 |
| McClure | 1981 | 10.1136/jcp.34.10.1167 |
| Van Kruijsdijk | 2011 | 10.1161/CIRCHEARTFAILURE.111.962183 |
| Torfs | 2016 | 10.1161/CIRCULATIONAHA.115.019178 |
| Schellhammer | 2002 | [10.1016/j.jccase.2021.08.011](https://doi.org/10.1016%2Fj.jccase.2021.08.011) |
| Roberts | 1987 | [10.1016/0002-9149(87)90978-7](https://doi.org/10.1016/0002-9149(87)90978-7) |
| Rossi | 2003 | 10.1007/s00428-003-0816-0 |
| Chow | 1992 | 10.1111/j.1365-2559.1992.tb00414.x. |
| Barson | 1981 | PMID: **6264379** |
| Simonson | 2007 | 10.1097/RTI.0b013e31806ad289. |
| Itoh | 1997 | [10.1253/jcj.61.798](https://doi.org/10.1253/jcj.61.798) |
| Barnard | 1985 | PMID: **15226996** |
| Jing | 1998 | 10.2214/ajr.170.4.9530031 |
| Lim | 2021 | 10.1093/ehjcr/ytaa564 |
| Dederer | 2018 | 10.1093/ehjcr/yty126 |
| Tominaga | 2015 | [10.1007/s13730-014-0128-z](https://doi.org/10.1007/s13730-014-0128-z) |
| Cao | 2009 | [10.3941/jrcr.v3i2.114](https://doi.org/10.3941%2Fjrcr.v3i2.114) |
| Akbas | 2014 | 10.4172/2155-6148.1000409 |
| Ahmed | 2019 | [10.1136/bcr-2018-228054](https://doi.org/10.1136%2Fbcr-2018-228054) |
| Freeman | 2010 | 10.1016/j.jcct.2010.08.005 |
| Vermeulen | 2018 | 10.1016/j.jcct.2017.09.016 |
| Kunz | 2017 | [10.14302/issn.2641-5518.jcci-17-1509](https://doi.org/10.14302/issn.2641-5518.jcci-17-1509) |
| Ng | 2017 | 10.1093/ehjci/jex324. |
| Wada | 1993 | [10.1253/jcj.57.567](https://doi.org/10.1253/jcj.57.567) |
| Zaidi | 2005 | [10.1007/s00246-004-0765-7](https://doi.org/10.1007/s00246-004-0765-7) |
| Lapatto | 2000 | [10.1046/j.1365-2796.2000.00588.x](https://doi.org/10.1046/j.1365-2796.2000.00588.x) |
| Maiese | 2019 | <https://doi.org/10.1016/j.jflm.2019.05.004> |
| Aras | 2005 | [10.1007/s10554-005-9006-2](https://doi.org/10.1007/s10554-005-9006-2) |
| Kimura | 2019 | [10.2169/internalmedicine.2039-18](https://doi.org/10.2169%2Finternalmedicine.2039-18) |
| Sui | 2018 | [10.1097/MD.0000000000013582](https://doi.org/10.1097%2FMD.0000000000013582) |
| Catellier | 1990 | [10.1002/clc.4960130410](https://doi.org/10.1002/clc.4960130410) |
| Terman | 1970 | 10.1016/0002-9343(71)90182-3. |
| Katz | 1988 | [10.1016/0002-9343(88)90517-7](https://doi.org/10.1016/0002-9343(88)90517-7) |
| Moltz | 2001 | [10.1097/00008480-200108000-00015](https://doi.org/10.1097/00008480-200108000-00015) |
| Schuster | 1992 | [10.1007/BF00838784](https://doi.org/10.1007/bf00838784) |
| de Moraes | 1986 | [10.1007/BF02386824](https://doi.org/10.1007/bf02386824) |
| Rayner | 1986 | [10.1136/bmj.293.6557.1277-a](https://doi.org/10.1136/bmj.293.6557.1277-a) |
| Koshy | 1975 | [10.1056/nejm197501232920414](https://doi.org/10.1056/nejm197501232920414) |
| Kapandji | 2018 | [10.1097/CCM.0000000000003130](https://doi.org/10.1097/ccm.0000000000003130) |
| Fierer | 1970 | <https://doi.org/10.1016/0002-9149(70)90741-1> |
| Kloeppel | 2001 | [10.1097/00004728-200105000-00013](https://doi.org/10.1097/00004728-200105000-00013) |
| Pak | 2018 | 10.4103/JCPC.JCPC_29_17 |
| Testelli | 1964 | [10.1016/0002-9149(64)90086-4](https://doi.org/10.1016/0002-9149(64)90086-4) |
| Kirk | 1966 | [10.1136/hrt.28.3.342](https://doi.org/10.1136/hrt.28.3.342) |
| Macieira-Coelho | 1975 | 10.1177/000331977502601005 |
| Roberts | 1981 | 10.1016/0002-9343(81)90163-7 |
| Matsui | 2012 | 10.1159/000343497. |
| Nakagawa | 2005 | [10.5414/cnp63313](https://doi.org/10.5414/cnp63313) |
| Martín-Cuartero | 2007 | [10.1157/13111241](https://doi.org/10.1157/13111241) |
| Shackley | 2011 | [10.1016/j.carpath.2010.04.004](https://doi.org/10.1016/j.carpath.2010.04.004) |
| Patterson | 1974 | [10.1136/thx.29.5.589](https://doi.org/10.1136/thx.29.5.589) |
| Ernestene | 1951 | [10.1161/01.CIR.3.5.690](https://doi.org/10.1161/01.CIR.3.5.690) |
| Edelstein | 1946 | [10.1016/0002-8703(46)90434-6](https://doi.org/10.1016/0002-8703(46)90434-6) |
| Finestone | 1949 | <https://doi.org/10.1093/ajcp/19.10.974> |
| Topaz | 1986 | [10.1007/BF02328954](https://doi.org/10.1007/bf02328954) |
| Canesin | 1999 | [10.1590/s0066-782x1999001200004](https://doi.org/10.1590/s0066-782x1999001200004) |
| El Bialy | 2005 | 10.1177/107424840501000208. |
| Lasser | 1983 | <https://doi.org/10.1016/S0046-8177(83)80305-0> |
| Lasky | 1954 | [10.7326/0003-4819-40-3-626](https://doi.org/10.7326/0003-4819-40-3-626) |
| Findlay | 2019 | [10.1155/2019/6707690](https://doi.org/10.1155%2F2019%2F6707690) |
| Butz | 2010 | [10.1093/ejechocard/jep148](https://doi.org/10.1093/ejechocard/jep148) |
| Kempf | 2009 | [10.3941/jrcr.v3i2.114](https://doi.org/10.3941%2Fjrcr.v3i2.114) |
| Minomo | 2021 | 10.1186/s13256-020-02588-2. |
| Singh | 2021 | 10.1007/s10554-020-01972-9. |
| Washino | 2020 | doi: [10.18999/nagjms.82.4.775](https://doi.org/10.18999%2Fnagjms.82.4.775) |
| Lippolis | 2021 | [10.1016/j.jccase.2020.10.013](https://doi.org/10.1016%2Fj.jccase.2020.10.013) |
| Kuchynka | 2020 | [10.1093/ehjci/jeaa248](https://doi.org/10.1093/ehjci/jeaa248) |
| Hu | 2020 | [10.1148/ryct.2020190204](https://doi.org/10.1148/ryct.2020190204) |
| Glick | 2020 | [10.1136/postgradmedj-2019-137211](https://doi.org/10.1136/postgradmedj-2019-137211) |
| Duarte | 2020 | [10.36660/abc.20190146](https://doi.org/10.36660%2Fabc.20190146) |
| Seo | 2018 | doi: [10.3346/jkms.2018.33.e162](https://doi.org/10.3346%2Fjkms.2018.33.e162) |
| Nijjar | 2018 | [10.1093/ehjci/jey204](https://doi.org/10.1093/ehjci/jey204) |
| Na | 2018 | 10.1007/s12024-017-9936-8. |
| Cecconi | 2016 | <https://doi.org/10.1093/eurheartj/ehw545> |
| Lopes | 2015 | [10.1016/j.repc.2014.10.003](https://doi.org/10.1016/j.repc.2014.10.003) |
| Halpern | 2015 | 10.1111/chd.12243. |
| Diez-delhoyo | 2015 | <https://doi.org/10.1093/ehjci/jev026> |
| Buchner | 2015 | [10.1093/ehjci/jev107](https://doi.org/10.1093/ehjci/jev107) |
| Revilla | 2012 | [10.1093/ehjci/jes037](https://doi.org/10.1093/ehjci/jes037) |
| Austin | 2013 | [10.1016/j.jcct.2012.07.004](https://doi.org/10.1016/j.jcct.2012.07.004) |
| Lee | 2012 | [10.4250/jcu.2012.20.4.193](https://doi.org/10.4250%2Fjcu.2012.20.4.193) |
| Jakl | 2010 | [10.14712/18059694.2016.83](https://doi.org/10.14712/18059694.2016.83) |
| Ionescu | 2009 | [10.1007/BF03086310](https://doi.org/10.1007%2FBF03086310) |
| Al Senaidi | 2009 | 10.1007/s00246-009-9443-0 |
| Lee | 2012 | [10.4250/jcu.2012.20.4.193](https://doi.org/10.4250%2Fjcu.2012.20.4.193) |
| Tom | 2006 | <https://doi.org/10.4065/81.3.335> |
| Mullens | 2006 | [10.1136/hrt.2005.068163](https://doi.org/10.1136%2Fhrt.2005.068163) |
| Robles | 2005 | 10.1007/s10554-006-9196-2 |
| Al-daraji | 2005 | [10.1111/j.1365-2559.2005.02202.x](https://doi.org/10.1111/j.1365-2559.2005.02202.x) |
| Fujimoto | 1990 | [10.1007/BF02550426](https://doi.org/10.1007/bf02550426) |
| Yoshihara | 2022 | [10.1016/j.jccase.2021.08.011](https://doi.org/10.1016%2Fj.jccase.2021.08.011) |
| Yokoi | 2022 | [10.1016/j.jccase.2021.07.010](https://doi.org/10.1016%2Fj.jccase.2021.07.010) |
| Kang | 2022 | 10.3348/jksr.2021.0181 |
| Tung | 2021 | [10.17161/kjm.vol14.15359](https://doi.org/10.17161%2Fkjm.vol14.15359) |
| Mitsui | 2021 | [10.1016/j.carpath.2020.107298](https://doi.org/10.1016/j.carpath.2020.107298) |
| Hankamp | 1956 | <https://doi.org/10.1148/68.4.564> |
| Canu | 2021 | 10.1016/j.jaccas.2021.04.037. |
| Chen | 2022 | [10.6515/ACS.202205_38(3).20211004A](https://doi.org/10.6515%2FACS.202205_38(3).20211004A) |
| Sedlock | 2019 | https://doi.org/10.29046/TMF.020.1.005 |
| Santos | 2019 | 10.1590/S1678-9946201961039. |
| Pejic | 2019 | 10.25259/JCIS_113_2019. |
| Monnier-Cholley | 2018 | [10.1007/s00134-018-5168-y](https://doi.org/10.1007/s00134-018-5168-y) |
| Furman | 2018 | [10.1016/j.jcct.2018.10.006](https://doi.org/10.1016/j.jcct.2018.10.006) |
| Torracchi | 2017 | DOI: [10.1016/j.arbr.2017.01.007](https://www.archbronconeumol.org/en-myocardial-calcification-a-rare-complication-articulo-S1579212917300113) |
| Salisbury | 2009 | [10.1016/j.jcct.2009.05.016](https://doi.org/10.1016/j.jcct.2009.05.016) |
| Tomaszuk-Kazberuk | 2012 | [10.1093/ejechocard/jer315](https://doi.org/10.1093/ejechocard/jer315) |
| Hewitt | 1947 | [10.1136/bmj.2.4536.959-a](https://doi.org/10.1136/bmj.2.4536.959-a) |
| Sinicina | 2005 | [10.1007/s00428-005-0022-3](https://doi.org/10.1007/s00428-005-0022-3) |
| Iwatani | 2009 | [10.1093/ndtplus/sfp018](https://doi.org/10.1093%2Fndtplus%2Fsfp018) |
| Chan | 2016 | [10.12809/hkjr1615347](http://dx.doi.org/10.12809/hkjr1615347) |
| Lin | 2014 | <https://doi.org/10.1016/j.healun.2013.10.027> |
| Hermann | 1963 | 10.1001/jama.1963.63710030036021a |
| Ito | 2015 | [10.2169/internalmedicine.54.4161](https://doi.org/10.2169/internalmedicine.54.4161) |
| Kaimoto | 2014 | [10.2169/internalmedicine.51.7323](https://doi.org/10.2169/internalmedicine.51.7323) |
| Cohnert | 1988 | PMID: **3049982** |
| Zakout | 2013 | https://doi.org/10.4021/jmc943w |
| Usui | 2019 | [10.1016/j.carpath.2019.07.005](https://doi.org/10.1016/j.carpath.2019.07.005) |
| Moudgil | 2020 | <https://doi.org/10.1161/CIRCIMAGING.119.009978> |
| Tu | 2020 | https://doi.org/10.1186/s12887-020-1973-x |
| Pardo-Mindán | 1986 | PMID: **2957472** |
| Somers | 1962 | [10.1136/hrt.24.3.324](https://doi.org/10.1136%2Fhrt.24.3.324) |
| Arndt | 2974 | [10.2214/ajr.122.1.133](https://doi.org/10.2214/ajr.122.1.133) |
| Shiga | 2011 | [10.2169/internalmedicine.50.5739](https://doi.org/10.2169/internalmedicine.50.5739) |
| Littman | 1963 | [10.1161/01.cir.28.5.938](https://doi.org/10.1161/01.cir.28.5.938) |
| Avila-Vanzzini | 2014 | <https://doi.org/10.1016/j.acmx.2013.06.001> |
| Bartrum | 1975 | PMID: **1158961** |
| Marciniak | 2015 | [10.1161/CIRCIMAGING.114.002750](https://doi.org/10.1161/circimaging.114.002750) |
| Koestner | 1978 | PMID: **580880** |
| Yeh | 2009 | [10.1111/j.1542-4758.2009.00353.x](https://doi.org/10.1111/j.1542-4758.2009.00353.x) |
| Illyes | 1989 | PMID: **2740142** |
| Arora | 1975 | PMID: **1138676** |
| Soni | 2008 | [10.1097/RLU.0b013e31817792e4](https://doi.org/10.1097/rlu.0b013e31817792e4) |
| Huhmann | 1977 | [10.1055/s-0028-1096681](https://doi.org/10.1055/s-0028-1096681) |
| Nikolaidou | 2020 | [10.1093/ehjci/jeaa121](https://doi.org/10.1093/ehjci/jeaa121) |
| Gerlis | 1999 | [10.1017/s1047951100005138](https://doi.org/10.1017/s1047951100005138) |
| L M Gerlis | 1994 | [10.1016/1054-8807(94)90036-1](https://doi.org/10.1016/1054-8807(94)90036-1) |
| Cesani | 1996 | [10.1007/BF02550969](https://doi.org/10.1007/bf02550969) |
| Nair | 1980 | PMID: **7228838** |
| Di Leo | 2003 | [10.5414/cnp59059](https://doi.org/10.5414/cnp59059) |
| Vainsel | 1970 | [10.1136/adc.45.240.254](https://doi.org/10.1136/adc.45.240.254) |
| Toniato | 2007 | [10.1111/j.1445-2197.2007.04239.x](http://dx.doi.org/10.1111/j.1445-2197.2007.04239.x) |
| Ahlgren | 2013 | [10.1111/echo.12031](https://doi.org/10.1111/echo.12031) |
| Okada | 2002 | [10.1034/j.1399-0012.16.s8.3.x](https://doi.org/10.1034/j.1399-0012.16.s8.3.x) |
| Ross | 1986 | [10.1016/0002-9149(86)90783-6](https://doi.org/10.1016/0002-9149(86)90783-6) |
| MacLean | 1995 | PMID: **7699668** |
| Fan | 2006 | <https://doi.org/10.1080/00313020600699193> |
| Hara | 2007 | [10.1016/j.carrev.2006.10.005](https://doi.org/10.1016/j.carrev.2006.10.005) |
| Matias Lopez Avecilla | 2020 | [10.1093/ehjcr/ytaa283](https://doi.org/10.1093/ehjcr/ytaa283) |
| Tomonobu Yanase | 2019 | [10.1002/jgf2.246](https://doi.org/10.1002%2Fjgf2.246) |
| Xacobe Flores-Ríos | 2013 | <https://doi.org/10.1016/j.rec.2011.11.017> |
| Samuel G Wittekind | 2017 | [10.1177/2324709617729393](https://doi.org/10.1177/2324709617729393) |
| Biplab Das | 2016 | [10.4103/0971-5916.198670](https://doi.org/10.4103%2F0971-5916.198670) |
| L. MINOR BLACKFORD | 1947 | [10.7326/0003-4819-27-6-1036](https://doi.org/10.7326/0003-4819-27-6-1036) |
| Milton C. Borman | 1943 | <https://doi.org/10.7326/0003-4819-18-5-857> |
| McMillan | 1945 | DOI: [10.1016/0002-9149(87)90978-7](https://doi.org/10.1016/0002-9149(87)90978-7) |
| F. M. Groedel | 1911 | - |
| Thomas Scholz | 1924 | 10.1001/archinte.1924.00120010035003 |
| T.W. DAVIDSON | 1928 | DOI: [10.1136/bmj.1.3501.212](https://doi.org/10.1136/bmj.1.3501.212) |
| Cohen | 1937 | 10.1001/archinte.1937.00180030103011 |
| Parkinson | 1938 | - |
| Hamid Jallal | 2022 | [10.11604/pamj.2022.41.8.32886](https://doi.org/10.11604%2Fpamj.2022.41.8.32886) |
| Joo-Young Na | 2018 | [10.1007/s12024-017-9936-8](https://doi.org/10.1007/s12024-017-9936-8) |
| Daiki Shako | 2021 | [10.1016/j.jccase.2021.10.002](https://doi.org/10.1016%2Fj.jccase.2021.10.002) |
| Lakshmi Muthukumar | 2016 | [10.1002/ehf2.12100](https://doi.org/10.1002/ehf2.12100) |
| Tadao Aikawa | 2019 | <https://doi.org/10.1093/eurheartj/ehz051> |
| Alfredo Renilla | 2015 | [10.1016/j.repc.2015.05.007](https://www.revportcardiol.org/pt-left-ventricular-calcification-in-patient-articulo-S0870255115002735) |
| C Tennstedt | 2000 | [10.1002/(sici)1097-0223(200004)20:4<287::aid-pd802>3.0.co;2-k](https://doi.org/10.1002/(sici)1097-0223(200004)20:4%3c287::aid-pd802%3e3.0.co;2-k) |
| Yonggang Yuan | 2019 | [10.1186/s12872-019-01305-2](https://doi.org/10.1186/s12872-019-01305-2) |
| Dany Debs | 2019 | [10.1016/j.jaccas.2019.05.009](https://doi.org/10.1016/j.jaccas.2019.05.009) |
| Roopali Khanna | 2016 | [10.4103/1995-705X.192557](https://doi.org/10.4103/1995-705x.192557) |
| Kook-Jin Chun | 2014 | [10.1080/ac.69.2.3017310](https://doi.org/10.1080/ac.69.2.3017310) |
| M A Rashwan | 1995 | [10.1136/hrt.73.3.284](https://doi.org/10.1136/hrt.73.3.284) |
| Antonio Grimaldi | 2012 | PMID: **22808844** |
| Weiliang Huang | 2019 | [10.1097/MD.0000000000016183](https://doi.org/10.1097%2FMD.0000000000016183) |
| Zhiqiang Han | 2020 | PMID: **32240101** |
| Cinzia D'Angelo | 2016 | [10.1714/2174.23492](https://doi.org/10.1714/2174.23492) |
| Dursun Aras | 2005 | [10.1007/s10554-005-9006-2](https://doi.org/10.1007/s10554-005-9006-2) |
| Masashi Kawamura | 2011 | [10.1111/j.1540-8191.2011.01364.x](https://doi.org/10.1111/j.1540-8191.2011.01364.x) |
| P F Fazzini | 1967 | PMID: **5606012** |
| Derward Lepley | 1974 | [10.1016/s0003-4975(10)64411-6](https://doi.org/10.1016/s0003-4975(10)64411-6) |
| I GORE | 1949 | PMID: **18152866** |
| Jones | 2014 | [10.12998/wjcc.v2.i5.142](https://doi.org/10.12998%2Fwjcc.v2.i5.142) |
| Sonoko Maemura | 2016 | doi: 10.1253/circj.CJ-16-0512 |
| Nydia Avila-Vanzzini | 2014 | DOI: [10.1016/j.acmx.2013.06.001](https://www.elsevier.es/es-revista-archivos-cardiologia-mexico-293-articulo-heart-calcification-idiopathic-cardiac-osseous-S1405994014000263) |
| M Lengyel | 1985 | [10.1016/0002-9149(85)91154-3](https://doi.org/10.1016/0002-9149(85)91154-3) |
| H Gzara | 2015 | [10.1097/MD.0000000000035823](https://doi.org/10.1097/md.0000000000035823) |
| Tansu Karaahmet | 2009 | [10.1111/j.1751-7133.2008.00031.x](https://doi.org/10.1111/j.1751-7133.2008.00031.x) |
| Jozef Krajcovic | 2013 | [10.1007/s00246-013-0637-0](https://doi.org/10.1007/s00246-013-0637-0) |
| Nora F Fnon | 2023 | [10.1016/j.legalmed.2023.102221](https://doi.org/10.1016/j.legalmed.2023.102221) |
| Ben Zhang | 2014 | [10.1017/S1047951113000073](https://doi.org/10.1017/s1047951113000073) |
| Marcela S Oliveira | 2011 | [10.1016/j.carpath.2010.01.012](https://doi.org/10.1016/j.carpath.2010.01.012) |
| Tomas Skala | 2019 | [10.5507/bp.2018.052](https://doi.org/10.5507/bp.2018.052) |
| W L Chan | 1987 | [10.1002/clc.4960100917](https://doi.org/10.1002/clc.4960100917) |
| G R Veldtman | 1999 | [10.1136/hrt.81.1.92](https://doi.org/10.1136%2Fhrt.81.1.92) |
| Kemal Baysal | 2008 | [10.1159/000151341](https://doi.org/10.1159/000151341) |
| Layla Rifai | 2011 | [10.1510/icvts.2010.259911](https://doi.org/10.1510/icvts.2010.259911) |
| Ninlapa Pruksanusak | 2014 | <https://doi.org/10.7863/ultra.33.10.1871> |
| C L Bose | 1983 | PMID: **6344839** |
| Ana Concheiro-Guisán | 2006 | [10.1080/15513810601015605](https://doi.org/10.1080/15513810601015605) |
| G L Winters | 1991 | PMID: **1924275** |
| L M Gerlis | 1994 | <https://doi.org/10.1016/1054-8807(94)90036-1> |
| K D Bolz | 1988 | [10.1055/s-2008-1048378](https://doi.org/10.1055/s-2008-1048378) |
| G Magnani | 1992 | PMID: **8521429** |
| O. Detry | 2002 | [10.1007/s00147-001-0364-y](https://doi.org/10.1007/s00147-001-0364-y) |
| F. Mookadam | 2010 | [10.1253/circj.cj-10-0107](https://doi.org/10.1253%2Fcircj.cj-10-0107) |
| P. Palka | 2001 | <https://doi.org/10.1161/hc2401.092123> |
| Vèlez-Roa | 2006 | <https://doi.org/10.1093/eurheartj/ehi871> |
| M N Lowenthal | 2000 | PMID: **10774283** |
| Josef Finsterer | 2007 | [10.1016/j.carpath.2006.12.005](https://doi.org/10.1016/j.carpath.2006.12.005) |
| J Kvaerness | 1991 | [10.1097/00004728-199105000-00028](https://doi.org/10.1097/00004728-199105000-00028) |
| G Vijayaraghavan | 1990 | PMID: **2093711** |
| I Steiner | 1985 | PMID: **4017035** |
| A K Pande | 2001 | PMID: **11225140** |
| L Goldberg | 2001 | PMID: **11717698** |
| Vedat Davutoglu | 2004 | [10.1097/01.crd.0000144102.76080.fd](https://doi.org/10.1097/01.crd.0000144102.76080.fd) |
| Jutta Karner | 2008 | [10.1016/j.hrtlng.2007.10.006](https://doi.org/10.1016/j.hrtlng.2007.10.006) |
| I Steiner | 1985 | PMID: **4017028** |
| Chiho Tokunaga | 2010 | [10.1016/j.athoracsur.2009.07.095](https://doi.org/10.1016/j.athoracsur.2009.07.095) |
| Francesco Sbrana | 2015 | [10.2459/JCM.0b013e32836277e0](https://doi.org/10.2459/jcm.0b013e32836277e0) |
| K Sommer | 1992 | [10.1378/chest.102.1.317](https://doi.org/10.1378/chest.102.1.317) |
| K K N Namboodiri | 2006 | [10.1136/hrt.2005.078667](https://doi.org/10.1136/hrt.2005.078667) |
| E Bertrand | 1969 | Title: [2 cases of endomyocardial fibrosis (with endocardial calcification in one of the cases)] |
| M Okamoto | 1988 | [10.1536/ihj.29.127](https://doi.org/10.1536/ihj.29.127) |
| Robert J. Siegel | 1987 | [10.1016/0002-9149(87)90274-8](https://doi.org/10.1016/0002-9149(87)90274-8) |
| E Mousseaux | 1966 | [10.1148/radiology.198.3.8628866](https://doi.org/10.1148/radiology.198.3.8628866) |
| Joy Li | 2021 | <https://doi.org/10.1148/ryct.2021200549> |
| Mariana Tinoco | 2021 | <https://doi.org/10.1093/ehjcr/ytab362> |
| Kenneth Hoang | 2020 | [10.1016/j.amjmed.2020.02.045](https://doi.org/10.1016/j.amjmed.2020.02.045) |
| Nydia Ávila-Vanzzin | 2014 | DOI: [10.1016/j.acmx.2013.06.001](https://www.elsevier.es/es-revista-archivos-cardiologia-mexico-293-articulo-heart-calcification-idiopathic-cardiac-osseous-S1405994014000263) |
| Maël Richard | 2023 | <https://doi.org/10.1016/S0140-6736(23)00682-7> |
| Raiza Pontes Rodrigues | 2022 | <https://doi.org/10.7326/aimcc.2022.0276> |
| Congjun Zeng | 2023 | [10.1016/j.jcct.2022.06.138](https://doi.org/10.1016/j.jcct.2022.06.138) |
| Sherif Moawad | 2022 | Title: Sepsis-induced Rapid Left Ventricular Calcification |
| Diego Xavier Chango Azanza | 2021 | [10.7759/cureus.18707](https://doi.org/10.7759/cureus.18707) |
| Al Senaidi | 2009 | [10.1007/s00246-009-9443-0](https://doi.org/10.1007/s00246-009-9443-0) |
| Mauricio Garcia-Cardenas | 2023 | [10.1016/j.hlc.2023.01.007](https://doi.org/10.1016/j.hlc.2023.01.007) |
| Banerjee, A. K.. | 2022 | covidwho-2179316 |
| Goel Vandana | 2023 | 10.35100/eurorad/case.17984 |
| F. BYLSMA | 1981 | [10.1007/BF03007262](https://doi.org/10.1007/bf03007262) |
| Soraya El Ghannudi | 2016 | [10.1016/j.ijcard.2016.07.146](https://doi.org/10.1016/j.ijcard.2016.07.146) |
| Kerem M. Vural | 2021 | [10.5152/AnatolJCardiol.2021.00748](https://doi.org/10.5152/anatoljcardiol.2021.00748) |
| Tatsuro Hitsumoto | 2015 | <https://doi.org/10.1016/j.cardfail.2015.08.116> |
| Robert R. Henderson | 1971 | [10.1056/NEJM197106032842208](https://doi.org/10.1056/nejm197106032842208) |
| Sui ML | 2021 | [10.12998/wjcc.v10.i13.4214](https://doi.org/10.12998%2Fwjcc.v10.i13.4214) |
| Rizwan Yusuf | 2022 | <https://doi.org/10.51253/pafmj.v72iSUPPL-3.9533> |
| Rajiv Ananthakrishna | 2016 | [10.1016/j.ihj.2016.02.016](https://doi.org/10.1016%2Fj.ihj.2016.02.016) |
| Susan Pumacayo-Cárdenas | 2020 | Ref: Pumacayo-Cárdenas S, Quea-Pinto E. Unusual Myocardial Calcification. J Clin Images. 2020;  3(1): 1049 |
| Abraham-Foscolo | 2022 | 10.36660/abc.20210370. |
| Hajdu | 1998 | [10.1002/(sici)1097-0223(199811)18:11<1186::aid-pd423>3.0.co;2-5](https://doi.org/10.1002/(sici)1097-0223(199811)18:11%3c1186::aid-pd423%3e3.0.co;2-5) |
| Buschmann | 2013 | [10.1007/s12024-012-9400-8](https://doi.org/10.1007/s12024-012-9400-8) |
| Barron | 2021 | [10.1177/1093526620970822](https://doi.org/10.1177/1093526620970822) |
| Holmes | 1964 | PMID: **14235561** |
| Duke | 1957 | PMID: **13434566** |
| Drut | 1998 | [10.1007/s100249900050](https://doi.org/10.1007/s100249900050) |
